# Prevalence and characteristics of *Listeria* species from selected African countries

**DOI:** 10.1186/s40794-021-00151-5

**Published:** 2021-09-15

**Authors:** Osman Adamu Dufailu, Muneer Oladipupo Yaqub, James Owusu-Kwarteng, Francis Addy

**Affiliations:** 1 Department of Veterinary Science, University for Development Studies, Tamale, Ghana; 2 Department of Microbiology, Usmanu Danfodiyo University, Sokoto, Nigeria; 3 Department of Food Science and Technology, University of Energy and Natural Resources, Sunyani, Ghana; 4 Department of Biotechnology, University for Development Studies, Tamale, Ghana

**Keywords:** *Listeria* spp., Antimicrobial resistance, Molecular epidemiology, Africa

## Abstract

Listeriosis, caused by *Listeria* spp., presents varying clinical manifestations among individuals, from moderate fecal infections such as diarrhea to severe infections such as septicemia, meningitis and abortion or newborn listeriosis in perinatal patients. In Africa, listeriosis is attributed to poor sanitation and cross-contamination in food processing environments, particularly ready to eat (RTE) foods including dairy products, leafy vegetables, fish and meat. Despite the global increase in reported cases and research on listeriosis, data from Africa remains scarce and this could lead to possible underestimation of the importance of listeriosis on the continent. This paper therefore presents a comprehensive overview of currently available reports on *Listeria* spp. in Africa with emphasis on molecular characteristics, antimicrobial susceptibility, and prevalence in food, animal and environmental samples. The majority of studies on *Listeria* spp. in Africa have so far focused on the prevalence and antibiotic susceptibility of *L. monocytogenes* isolated from RTE foods and raw meat but rarely from humans, animals, and the environment. The overall calculated average prevalence values from the available reports are 23.7 and 22.2% for *Listeria* spp. and *L. monocytogenes,* respectively. *Listeria* spp. isolated from different parts of Africa are generally sensitive to ciprofloxacin, but resistant to penicillin. The majority of these studies employed conventional culture and biochemical tests to characterize *Listeria* spp. However, the use of modern molecular techniques such as PCR and whole-genome sequencing is on the rise. Most of the studies employing molecular tools were carried out in South Africa and Nigeria, with the predominant strain reported in South Africa being ST6. In order to provide a better understanding of the importance of listeria in Africa, there is the need for extensive and coordinated studies using modern molecular-based techniques to characterize the various *Listeria* species, and to assess the disease epidemiology using the one health concept.

## Background

Listeriosis is predominantly a foodborne infection associated with microorganisms of the genus *Listeria*. *Listeria* mostly affects the elderly, immunocompromised individuals, pregnant women, and newborns [1, 2]. The World Health Organization (WHO) estimates the global burden of listeriosis to be 172,823 disability adjusted life years (DALYs) from 23,150 illnesses [3]. This estimate is based on several data sources from high-income and middle-income subregions, and arbitrary assumptions on regions with data gaps such as WHO African regions (WHO – AFRO) that had 3624 listeriosis incidences, 955 deaths and 27,045 DALYs.

*Listeria* spp. are gram-positive, catalase-positive bacteria that are motile at least at 30 °C, persist for long periods in the environment, and can grow in a wide range of temperatures. There are currently seventeen (17) identified *Listeria* species. However, *L. ivanovii* and *L. monocytogenes* remain the most important species that cause listeriosis in animals and humans [4, 5]. These species are globally distributed with varying prevalence rates in the different regions. Currently, a comprehensive review on listeriosis in Africa is unavailable, except for very few isolated reports [3]. Thus, the epidemiology of listeriosis in Africa is less understood, and can lead to underestimation of the disease burden on the continent. Meanwhile, a comprehensive overview of the existing published data on epidemiology, prevalence, antimicrobial resistance, and molecular characteristics of *Listeria* spp. from various countries would provide an outlook of the disease in Africa and highlight areas of urgent research needs.

In this narrative review, we have presented, in context, the currently available literature on the prevalence and molecular diversity of *Listeria* spp. in Africa. In addition, the resistance of *Listeria* spp. to common antibiotics, major epidemiological hypotheses and areas of urgent research needs are highlighted. All observational studies reporting on the prevalence, antimicrobial resistance/susceptibility, or characterization of *Listeria* species in Africa were searched on PubMed, Web of Science, EMBASE, Google Scholar and the Cochrane Library for this review.

## Epidemiology

**Fig. 1:**
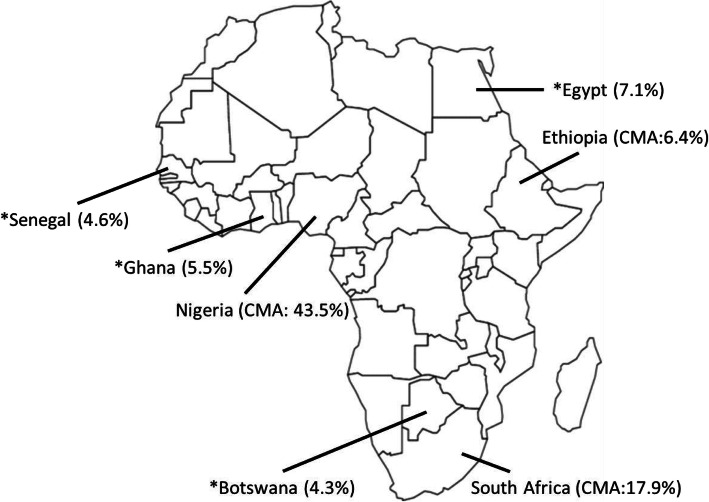
Prevalence of *Listeria monocytogenes* in selected African countries. *Average from single study; CMA: Calculated Mean Average (See Table 1.); Calculated Mean Average (CMA) = (Sum of prevalence rates/number of prevalence) × 100

The epidemiology of listeria in Africa is often marked by sporadic cases or major outbreaks. The 2017/2018 South African listeriosis outbreak awakened the world to the possible wide-spread of the disease in Africa [6, 7]. Humans risk multiple exposures to infection via contact with carriers of listeria. Listeria, the causative agent for listeriosis, can colonize up to 5% of healthy adults [7]. Adults with invasive listeriosis can present symptoms such as fever, stillbirth, and convulsion among others. The risk of infection is further exacerbated by the ubiquitous nature of the pathogen, making it difficult to get rid of listeriosis [4]. In Africa, *Listeria* spp., particularly *L. monocytogenes*, has been isolated from various food, animal and environmental sources in countries such as Nigeria, South Africa, Ghana, Ethiopia, Egypt and Botswana. Table 1 summarizes the prevalence of *Listeria* spp. in various food and environmental samples from different African countries (Fig. 1).

**Table 1:** Prevalence of *Listeria* spp. in food, environmental and human samples in Africa

Country	Sample Source	Isolated species	Sample size	Prevalence (%) (positive sample/sample size)	Reference
South Africa	Meat	*L. monocytogenes*	2017	14.7	[8]
South Africa	Irrigation water	*L. monocytogenes*	57	16.0	[9]
South Africa	Agricultural soil	*L. monocytogenes*	39	23.0	[9]
Nigeria	Raw beef and pork	*Listeria* spp*.*	100	19.0	[10]
Nigeria	Chicken flocks and meat	*L. monocytogenes*	426	91.8	[11]
Nigeria	Chicken meat/Pork/Beef	*Listeria* spp*.*	432	28.7	[12]
Nigeria	Raw meat and meat product	*Listeria* spp*.*	300	28.3	[13]
Nigeria	Salad vegetables, Salad and coleslaw	*L. monocytogenes*	355	3.9	[14]
Nigeria	Cabbage/Carrot/Cucumber/Lettuce/ Tomatoes	*L. monocytogenes*	555	44.0	[15]
Nigeria	Soft cheese	*L. monocytogenes*	116	49.1	[12]
Nigeria	Raw beef, pork and chicken	*L. monocytogenes*	205	7.8	[16]
Nigeria	Raw beef and chevon	*L. monocytogenes*	104	64.4	[5]
Ghana	Raw cow milk/Nunu/Boiled milk	*L. monocytogenes*	254	5.5	[17]
Ethiopia	Beef, butchers and restaurant	*L. monocytogenes*	450	4.4	[18]
Ethiopia	Pizza, cake, ice cream, minced beef, fish, raw meat, and unpasteurized milk	*L. monocytogenes*	384	6.3	[19]
Ethiopia	Vaginal samples of pregnant women	*L. monocytogenes*	141	8.5	[20]
Botswana	Cheese, raw milk, meat (biltong), frozen cabbage and salad	*L. monocytogenes*	1324	4.3	[21]
Egypt	Frozen chicken leg/Minced frozen beef/Frozen chicken fillet/Luncheon	*Listeria* spp*.*	100	43.0	[22]
Egypt	Stool culture	*L. monocytogenes*	28	7.1	[22]
Senegal	Vaginal samples of women who experienced miscarriage	*L. monocytogenes*	43	4.6	[23]

To date, the listeriosis outbreak in South Africa remains the largest in the world with over 1000 laboratory-confirmed cases and over 200 fatalities [24]. This unprecedented outbreak has been attributed to changes in food production and distribution processes in South Africa [25]. Before this outbreak, a marked increase in listeriosis cases was recorded in June 2017 with the source of infection being RTE processed meat product [25]. Following identification of the source of the outbreak, recalls of the affected products were made in South Africa and 15 other African countries [26]. Other reports from South Africa described the presence of *L. monocytogenes* in various food items [27]. In 2015, *L. monocytogenes* ST6 affecting humans were reported in Western Cape Province of South Africa [25]. From 2014 to 2016, the overall prevalence of *L. monocytogenes* in meat and meant products in South Africa was reported to be 14.7% (296/2017), with meats from local markets and ports of entry recording prevalence rates of 15.0% (264/1758) and 12.4% (32/259), respectively [8]. On the other hand, the prevalence of *L. monocytogenes* recovered from irrigation water and agricultural soil samples in South Africa were 6.8% (8/117) and 6.6% (12/183), respectively [9]. Other studies have also reported that municipal wastewater effluent is a possible reservoir and transporter of pathogenic *Listeria* in South Africa and this is of public health concern [28]. Taking into account that approximately 77% of South Africans depend on surface water for their daily activities, the reported prevalence of *Listeria* in irrigation water and agricultural soil could be potential source for infections [29]. From available data, the calculated mean prevalence of *L. monocytogenes* in South African is 17.9%. Despite the few recent reports on the prevalence of listeriosis in South Africa, comprehensive historic data on prevalence, epidemiology and outbreaks associated with *L. monocytogenes* is still lacking [25].

In Northern Africa, *Listeria* spp. was detected in 32% of luncheon, 32% of minced frozen beef, 52% of frozen chicken leg and 56% of frozen chicken fillet in Assiut, Egypt [22]. Additionally, the incidence of *Listeria* spp. in stool samples of children with underlying health conditions at Assiut University hospital was reported to be 7.14% (2/28). The data demonstrate a potential infection linked to consumption of listeria-containing meat and chicken products in Assiut city, Egypt [22].

In East Africa, the prevalence rate of *L. monocytogenes* among pregnant women in northern Ethiopia was reported to be 8.5% [20]. Also, 4.4% prevalence of *L. monocytogenes* was reported in beef and fomites [18]. In another study, a 26.1% prevalence of *Listeria* spp. was reported in food of animal origin [30]. Furthermore, a prevalence of 25 and 6.25% for *Listeria* species and *L. monocytogenes,* respectively, was reported from ready-to eat food of animal origin in Ethiopia [19].

In an investigation to assess the occurrence of *L. monocytogenes* in Gaborone, Botswana in Central Africa, Morobe et al., [21] analyzed food samples collected from five geographical areas. In their report, the overall prevalence of *L. monocytogenes* was 4.3% (57/1324). Out of the 57 *L. monocytogenes* isolates, 12.3% were derived from cheese while the highest *L. monocytogenes* prevalence of 33.3% was recorded in Gaborone South [21].

In West Africa, the Nigerian meat industry has been implicated in the transmission of *Listeria* spp. [10]. In Rivers State, South-South Nigeria, 7% prevalence of *L. monocytogenes* in raw meat samples was reported [10] while 91.8% prevalence of *L. monocytogenes* in chicken flocks and meat was reported in Oyo state, Nigeria [11]. In Lafia, Nigeria, the reported prevalence of *Listeria* spp. in beef and chevon were 58.2% (78/134) and 41.8% (56/134), respectively. Of these isolates, 64.4% (67/104) were confirmed to be *L. monocytogenes* [5]. In Enugu state, Nigeria, the frequency of *Listeria* spp. isolated from chicken meat, pork, and beef samples was 27.1, 13.2 and 45.8% respectively [12], while in Zaria, Nigeria, the prevalence of *L. monocytogenes* in raw meat and meat products was 4.0% [13]. Other studies in Nigeria reported the prevalence of *L. monocytogenes* in vegetables including in cabbage, carrot, cucumber, lettuce, and tomatoes to be, 28.28, 9.02, 23.36, 19.67, and 19.67%, respectively [15]. Additionally, the prevalence of *Listeria* spp. in locally made soft cheeses (wara) was 78%. Out of which 12.4% were identified as *L. monocytogenes* [31]. From this review, the calculated mean average prevalence of *L. monocytogenes* for Nigeria is 43.5%.

In Ghana, data on the prevalence of *L. monocytogenes* remains scarce. However, 5.5% (14/254) prevalence of *L. monocytogenes* in traditional milk products was reported [17]*.* Individual prevalence for raw cow milk, boiled milk and spontaneously fermented milk (*nunu*) were 8.8% (10/114), 0% (0/114), and 13.1% (11/84), respectively [17]. Additionally, *Listeria* was identified in fresh milk [32] and smoked fish [33]. *Listeria* spp. was not recorded when pregnancy related infections in rural communities was investigated [34].

## Antimicrobial resistance

Antimicrobial resistance has become a global public health issue. Several studies across Africa have reported the antimicrobial resistance profiles of *Listeria* species, specifically *L. monocytogenes*. Table 2 summarizes reported studies on antibiotic resistance profiles of listeria isolates in Africa.

**Table 2:** Antimicrobial susceptibility profile of *Listeria* spp. isolated in Africa

State/Country	Sample source	Organism	Resistant Antibiotics	Sensitive Antibiotics	Reference
Ethiopia	^a^RTE/Meat	*L. monocytogenes*	Penicillin and Nalidixic acid	Amoxicillin, Gentamicin, Vancomycin, Cephalothin, Cloxacillin and Sulfamethoxazole	[19]
Botswana	RTE/Meat	*L. monocytogenes*	Penicillin G	Fusidic acid, Erythromycin, Methicillin and Ampicillin	[21]
Ethiopia	Pregnant women	*L. monocytogenes*	Clindamycin, Penicillin G and Vancomycin	Erythromycin and Ciprofloxacin	[20]
South Africa	Environment/waste water	*Listeria spp.*	Penicillin, Nalidixic acid and Erythromycin	Amikacin, Gentamicin, Meropenem, Clindamycin, Ciprofloxacin, Streptomycin, Chloramphenicol	[35]
South Africa	RTE/Meat	*L. monocytogenes*	Streptomycin, Clindamycin and Fusidic acids	Ampicillin, Kanamycin and Amikacin	[8]
South Africa	Environment/soil and water	*L. monocytogenes*	Tetracycline, Doxycycline, Penicillin, and Erythromycin	Ampicillin, Gentamicin, Amikacin	[9]
Nigeria	RTE/Meat	*L. monocytogenes*	Penicillin, Cephalothin, Gentamicin, and Ciprofloxacin	ND	[12]
Nigeria	RTE/Meat	*Listeria spp.*	Ampiclox (ampicillin/cloxacillin) and Amoxicillin	Ciprofloxacin and Septrin (co-trimoxazole)	[31]
Nigeria	RTE/Meat	*L. monocytogenes*	Streptomycin and Sparfloxacin	Rifampicin	[5]
Nigeria	RTE/Meat/chicken flock	*L. monocytogenes*	Ampicillin-cloxacillin and Cefuroxime	Amoxicillin clavulanate	[11]
Nigeria	Beef/Pork/Chicken	*L. monocytogenes*	Amoxicillin, Tetracycline, Cloxacillin and Augmentin	Gentamicin, Erythromycin	[16]
Nigeria	RTE/Meat/fomite	*L. monocytogenes*	Augmentin, Erythromycin, Tetracycline and Rifampicin	Chloramphenicol, Gentamicin, Ampiclox and Clotrimoxazole	[10]
Ghana	Cow milk “Nunu”	*L. monocytogenes*	Neomycin	Amoxicillin, Ampicillin, Erythromycin, Gentamycin, Penicillin, Rifampicin, and Vancomycin	[17]

^a^*RTE* Ready to eat foods (meat, vegetable, Milk). *ND* Not determined

In Ethiopia, resistance of *L. monocytogenes* was reported for the following antibiotics: penicillin 66.7% (16/24), nalidixic acid 50% (12/24), tetracycline 37.5% (9/24) and chloramphenicol 16.6% (4/24). Furthermore, 16.6% were found to be multi-drug resistant [19]. In a different study, *L. monocytogenes* isolated from pregnant women in Ethiopia showed that isolates were resistant to clindamycin (66.7%), penicillin G (66.7%), vancomycin (50%) and amoxicillin (50%). However, isolates were sensitive to erythromycin (75%), ciprofloxacin (75%), trimethoprim/sulphamethaxazole (66.7%) and chloramphenicol (60%) [20]. Whereas, *Listeria spp*. isolated from wastewater treatment plants in Durban, South Africa, demonstrated 100% resistance to penicillin, nalidixic acid and erythromycin. Furthermore, these isolates were also resistant to ampicillin (83.33%), trimethoprim (67.95%), nitrofurantoin (64.10%) and cephalosporin (60.26%) [35].

Similarly, *L. monocytogenes* isolates recovered from irrigation water and agricultural soil from Eastern Cape Province, South Africa, were resistant to tetracycline (90%), doxycycline (85%), penicillin (80%), cefotaxime (80%), chloramphenicol (70%), linezolid (65%), erythromycin (60%) and trimethoprim/sulfamethoxazole (55%) [9]. The isolates were also reported to harbor *tetA*, *tetB*, *tetC*, *sulI*, *sulII*, *aadA*, *aac (3)-IIa* resistance genes and extended-spectrum beta-lactamase (ESBLs) including *blaTEM*, *blaCTX-M* group 9, *blaVEB* as well as *ampC*. However, none of the isolates carried the carbapenemase resistance genes [9].

In South Africa, another study reported that 1.7% *L. monocytogenes* isolated from meat showed multiple resistance to 13 of the 19 test antibiotics. Resistance was recorded for streptomycin (99.0%), clindamycin (97.3%), fusidic acids (95.6%), nitrofurantoin (79.7%), and gentamycin (74.4%). However, the isolates were sensitive to ampicillin (85.6%), kanamycin (84.6%), amikacin (77.6%), vancomycin (74.2%), and tetracycline (62.5%) [8].

In Botswana, *L. monocytogenes* isolated from RTE/meat showed multiple resistance against penicillin G (42.1%), sulphamethaxozole/trimethoprim (29.8%), chloramphenicol (28.3%), and tetracycline (22.8%). Resistance against penicillin G and tetracycline was a common pattern in all *L. monocytogenes* isolated from food products. However, the isolates were generally susceptible to fusidic acid, erythromycin, methicillin, ampicillin and cephalothin [21].

In Enugu State, South-East Nigeria, *L. monocytogenes* isolated from beef, chicken, and pork were examined against penicillin, cephalothin, amoxicillin, ampicillin, nitrofurantoin, vancomycin, tetracycline, gentamicin (aminoglycosides), gentamicin (macrolides), ciprofloxacin, sulphamethoxazole/trimethoprim and rifampicin. All *L. monocytogenes* showed 100% resistance against penicillin. Interestingly, only *L. monocytogenes* recovered from pork showed 100% resistance to more than one antibiotic, i.e., penicillin, cephalothin, sulphamethoxazole/ trimethoprim and ciprofloxacin [12]. The high and multidrug resistance of isolates from pork is a public health concern and could be attributed to the misuse of antibiotics in pig farming. Also, *L. monocytogenes* isolated from beef, pork and chicken recorded resistance to amoxicillin, tetracycline, augmentin and cloxacillin but sensitivity to erythromycin and gentamicin [16]*.*

In Ekiti, South-West Nigeria, *Listeria* spp. isolated from soft cheese (*wara*) showed 90 and 89% resistance against ampiclox (ampicillin/cloxacillin) and amoxicillin, respectively. However, the isolates were susceptible to ciprofloxacin and septrin (co-trimoxazole) [31]. *L. monocytogenes* isolated from beef and chevon in North-Central Nigeria were reported to be resistant to streptomycin (58.2%), sparfloxacin (55.2%), ampicillin (34.3%), and gentamicin (20.9%) [5]. *L. monocytogenes* has also been reported to be resistant to ampicillin and erythromycin, but susceptible to gentamicin and ciprofloxacin [14]. In another study, *L. monocytogenes* strains were resistant (100%) to both ampicillin-cloxacillin and cefuroxime but susceptible to amoxicillin clavulanate (86.1%) ciprofloxacin (43.8%), cloxacillin (36.1%), ceftriaxone (32.5%), gentamicin sulphate (27.8%), streptomycin sulphate (25.0%), pefloxacin (17.5%), erythromycin 5 μg (16.7%), co-trimoxazole (12.5%), erythromycin 10 μg (12.5%), and amoxicillin (6.3%) [11]. Similarly, 100% resistance to augmentin, erythromycin, tetracycline, rifampicin, and cloxacillin was recorded, with some isolates demonstrating a varying degree of resistance to norfloxacin (57.2%), levofloxacin (71.4%), and ciprofloxacin (71.4%). On the contrary, all *L. monocytogenes* isolates from retail meats were (100%) susceptible to chloramphenicol, gentamicin, ampiclox, clotrimoxazole, and streptomycin [10].

In Ghana, *L. monocytogenes* resistance against neomycin (61.3%) and tetracycline (24.2%) was observed. While intermediate susceptibilities were recorded for chloramphenicol, ciprofloxacin, clindamycin, doxycycline, kanamycin, neomycin, streptomycin, and tetracycline, general susceptibility (100%) to amoxicillin, ampicillin, erythromycin, gentamycin, penicillin, rifampicin, and vancomycin was observed [17].

Although antibiotics remain the conventional protocol for the treatment of listeriosis, some studies have also shown the potential of plant extracts in listeriosis chemotherapy. The effect of plant triterpenes: 3β-hydroxylanosta-9,24-dien-21-oic acid, methyl-3β-hydroxylanosta-9,24-dien-21-oate and 3β-acetylursolic acid, against *L. monocytogenes, L. ivanovii and L. grayi species* was investigated. The triterpenes’ minimum inhibitory concentration (MIC) values ranged from 0.185 to 1.67 mg/ml while the minimum bactericidal concentration (MBC) determination assay revealed that the triterpenes were bacteriostatic against *Listeria* spp. [36]. In summary, listeria isolates from Africa are generally susceptible to ampicillin and ciprofloxacin. However, the emergence of multidrug resistant strains is of serious public health concern in Africa.

## Molecular characterization

Genomic studies have been employed to elucidate the global circulation of *L. monocytogenes* [37]. In Africa, the serotypes of circulating strains of *L. monocytogenes* are largely unknown [38]. In epidemiological studies, rapid detection of listeriosis outbreaks is often by phenotypic and molecular characterizations [39]. Subtyping of *L. monocytogenes* isolates is essential for epidemiological investigation and for identification of the source of contamination [1]. Table 3 provides a summary of molecular tools employed in the characterization of *Listeria* spp. in Africa.

**Table 3:** Molecular Techniques used for Listeria Characterization in Africa

Country	Molecular Techniques	Gene/Serogroup/Strain	Reference
South Africa	Multilocus Sequence Typing (MLST) Prokaryotic Genome Annotation Pipeline (PGAP)	ST6	[25]
South Africa			
South Africa	Genome Assembly Multilocus Sequence Typing (MLST)	ST6	[6]
South Africa	Whole Genome Sequencing	ST1, ST121, ST204, and ST876	[40]
	Average Nucleotide Identity (ANI) PHAge Search Tool Enhanced Release (PHASTER) Nucleotide Basic Local Alignment Search Tool (NCBI-BLASTn)	Serogroup 4b (lineage I) Serogroup 1/2a (lineage II)	
	Polymerase Chain Reaction (PCR)	*inlJ, ipa, inlB, inlC*, and *inlA*	
South Africa	Polymerase Chain Reaction (PCR)	*iap, actA*, and *plcA*	[35]
South Africa	Polymerase Chain Reaction (PCR)	*inlA*, *inlB*, *inlC*, *inlJ*, *actA*, *hlyA*, *plcA*, *plcB*, and *iap*	[9]
Nigeria	Multilocus Sequence Typing (MLST)	ST5, ST155, CT2050 and CT2051	[37]
Nigeria	Polymerase Chain Reaction (PCR)	*hlyA* and *iap*	[41]
Nigeria	Multiplex PCR assay (mPCR)	*prfA, inlA, hlyA, actA,* and *iap*	[42]
	16S rRNA - based Phylogenetic Analysis	NGA 34A, NGA 35A, NGA 41A, and NGA 38A	
Nigeria	Polymerase Chain Reaction (PCR)	*hylA*	[13]
Ghana	PCR amplification of 16S rRNA gene and listeriolysin O gene; Multiplex PCR for serotyping	1/2a-3a, 1/2b-3b-7, 4b-4d-4e and 1/2c-3c	[17]

Multilocus Sequence Typing (MLST) has been used to prove that the 2015 and 2017 listeriosis outbreaks in South Africa were mainly due to contamination of meat products by *L. monocytogenes* ST6 [25]. *L. monocytogenes* ST6 is often associated with high fatality cases. Whole-genome sequencing approach, MLST, was used to demonstrate that *L. monocytogenes* ST6 was the most common serotype of *L. monocytogenes* detected in human listeriosis cases in Western Cape Province in South Africa [6].

Four different STs (ST1, ST121, ST204, and ST876) belonging to lineage I (serogroup 4b) and lineage II (1/2a) were identified using whole genome sequencing (WGS) to characterize six *L. monocytogenes* isolated from RTE meat products in South Africa [40]. From their report, the majority of the serogroup 4b (lineage I) strains clustered together while two isolates of serogroup 1/2a (lineage II) were apart compared to the other strains using core genome and average nucleotide identity (ANI) phylogenetic analyses. Additionally, twenty-four different prophages were identified using PHAge Search Tool Enhanced Release (PHASTER) software [40]. The National Center for Biotechnology Information- Nucleotide Basic Local Alignment Search Tool (NCBI-BLAST) showed that the investigated *L. monocytogenes* strains shared some major virulence genes encoded in the pathogenicity islands 1 and 3 of listeria [40]. All isolates harbored resistant genes against food antiseptics. Overall, all strains of *L. monocytogenes* isolated from ready to RTE meat products showed similar resistance profiles against heavy metal, and antibiotic [40]. Polymerase chain reaction (PCR) was also employed to demonstrate that most of the *L. monocytogenes* isolates harbored *inlJ* (*98.7*%*)* and *ipa* (95.6%*)* genes with at least one other internalin genes (*inlB, inlC*, or *inlA*) [40]. Using PCR, 26.92% of *Listeria* spp. isolated from the effluent of wastewater in Durban, South Africa, were found to contain virulence genes, with 14.10, 5.12, and 21% harboring the *actA*, *plcA* and *iap* genes, respectively. Additionally, the study employed enzymatic hydrolysis to characterize gelatinase, protease, and haemolysin [35]. Similarly, PCR was used to confirm that all *L. monocytogenes* isolates recovered from irrigation water and agricultural soil in Eastern Cape Province, South Africa harbored nine virulence genes (*inlA*, *inlB*, *inlC*, *inlJ*, *actA*, *hlyA*, *plcA*, *plcB*, and *iap)* [9].

In Nigeria, the first *L. monocytogenes* genome sequence was of three isolates recovered from fresh leaves and vegetables in South-Eastern Nigeria. The size of the draft genome was between 2.93 to 3.06 Mb, with 37.9% GC content. Isolates were found to belong to ST155 and ST5 using MLST, while CT2050 and CT2051 were defined as new cgMLST types [37]. In South-East Nigeria, 23(30.67%) and 41(54.67%) *Listeria* spp. were isolated from a cereal-based food (fura), and a RTE fermented milk (nunu), respectively. Using PCR, the isolates were classified as human pathogenic serogroup 1/2a and 4b, with some strains harboring virulence genes *hlyA* and *iap* [41]. Similarly, *L. monocytogenes* isolates from raw meat and meat products in Kaduna, Nigeria was characterized using uniplex PCR to detect the *hylA* gene [13].

Multiplex PCR was employed to identify virulence-associated genes *(prfA, inlA, hlyA, actA,* and *iap)* in *L. monocytogenes* from milk in Nigeria [42]. The study further compared the 16S rRNA sequence of the isolates to the reference *L. monocytogenes* ATCC 19155 and a phylogenetic analysis enabled the clustering of the isolates into two lineages; lineage A (responsible for epidemic listeriosis) and lineage B (responsible for sporadic cases of listeriosis). The categorization of the *L. monocytogenes* isolates into the two lineages provides better overview of potential risk of listeriosis outbreak by these isolates [42]. The Nigerian *L. monocytogenes* isolates (NGA 34A, NGA 35A, and NGA 41A) were phylogenetically closer to J1776; N1-011A; R2–502; J1816; and J2–031, whereas isolate (NGA 38A) was closer to EDG; J1–220; J1926; J1817; and J2–1091 [42].

In Ghana, multiplex PCR was used to identify virulence-associated genes, *plcA*, *actA*, *hlyA*, *iap* and *prfA*, as well as *inlA*, *inlB*, *inlC*, and *inlJ* in *L. monocytogenes* isolates from raw and fermented cow milk product [17]. The presence of single or multiple genes enable categorization of isolates into serogroups 1/2a-3a (32/62, 51.6%), 1/2b-3b-7 (14/62, 22.6%), 4b-4d-4e (9/62, 14.5%) and 1/2c-3c (7/62, 11.3%) [17].

In summary, PCR techniques have been used in a few studies to identify and characterize the virulence potential of *L. monocytogenes* in Africa. However, the use of whole-genome sequencing to enhance characterization is gradually emerging in Africa, particularly in South Africa and Nigeria.

## Conclusion and future perspective

*Listeria* spp., particularly *L. monocytogenes,* is generally reported to have a low prevalence rate but a high fatality rate. The possible explanations for the epidemiological differences of *Listeria spp.* across Africa could be attributed to the variation in study groups, sampling source, microbial diversity, and geographic location. Currently, information on the prevalence, antimicrobial susceptibility profiles, and molecular characteristics of *Listeria* spp. in Africa is limited and disconnected. Here the highest calculated mean average of *Listeria* spp. was recorded in Nigeria, followed by South Africa. Although, *L. monocytogenes* isolates are generally susceptible to many antibiotics, the observed single and multiple antibiotics resistant strains detected are a cause for concern. Our review shows that, the majority of the African *Listeria* isolates are resistant to penicillin, erythromycin, tetracycline, and amoxicillin, while most isolates are susceptible to gentamicin, amikacin, ampicillin and ciprofloxacin.

While studies on the prevalence and antibiotic susceptibility profile of *Listeria* spp. are considered limited globally, molecular characterization of *Listeria* spp. in Africa remains very scarce. Genomic sequences are often employed to decipher population structure, pathogen evolution and transmission networks. Although there has been an increase in the use of PCR and MLST to study listeriosis since 2018, most of the studies were limited to *L. monocytogenes* isolates from RTE foods in South Africa and Nigeria.

To develop effective preventive and control measures against potential *Listeria* spp. outbreaks, it is crucial to actively research the molecular epidemiology of listeriosis. To understand the dynamics of listeria infection in Africa, other African countries should contribute to studies aimed at a complete overview of the prevalence of listeria in Africa. There is also the need to elucidate the link between the epidemiological patterns of *Listeria* spp. from various sources (animals, humans and environment), by adapting the one health concept to study not only *L. monocytogenes* in RTE food but also study other *Listeria* spp. in humans, animals, and the environment.

## Data Availability

Not applicable.

## Abbreviations

L: Listeria
Spp: species (spp)
RTE: Ready to Eat
WHO: World Health Organization
DALYs: Disability adjusted life daily
AFRO: African regions
CMA: Calculated mean Average
ND: Not determined
ESBL: Extended-spectrum beta-lactamase
MIC: Minimum inhibitory concentration
MBC: Minimum bactericidal concentration
PGAP: Prokaryotic Genome Annotation Pipeline
RNA: Ribonucleic acid
ANI: Average nucleotide identity
PHASTER: PHAge Search Tool Enhanced Release
NCBI-BLASTn: National Center for Biotechnology Information-NucleotideBasic Local Alignment Search Tool
PCR: Polymerase chain reaction
MLST: Multilocus Sequence Typing
ST(s): Serotype(s)
WGS: Whole Genome Sequencing (WGS)
